# A Novel Mechanism of Action of Histone Deacetylase Inhibitor Chidamide: Enhancing the Chemotaxis Function of Circulating PD-1(+) Cells From Patients With PTCL

**DOI:** 10.3389/fonc.2021.682436

**Published:** 2021-06-01

**Authors:** Chong Wei, Shaoxuan Hu, Mingjie Luo, Chong Chen, Wei Wang, Wei Zhang, Daobin Zhou

**Affiliations:** ^1^ Department of Hematology, Peking Union Medical College Hospital, Chinese Academy of Medical Sciences & Peking Union Medical College, Beijing, China; ^2^ Key Laboratory of Carcinogenesis and Translational Research (Ministry of Education), Department of Lymphoma, Peking University Cancer Hospital & Institute, Beijing, China; ^3^ Department of General Surgery, Peking Union Medical College Hospital, Chinese Academy of Medical Sciences & Peking Union Medical College, Beijing, China; ^4^ Department of Immunology, Institute of Basic Medical Sciences, Chinese Academy of Medical Sciences; School of Basic Medicine, Peking Union Medical College, Beijing, China

**Keywords:** peripheral T-cell lymphoma, chidamide, innate immune, chemotaxis, programmed cell death-1

## Abstract

**Background:**

Peripheral T‐cell lymphomas (PTCLs) are a heterogeneous group of neoplasms characterized by a poor prognosis. Histone deacetylase (HDAC) inhibitors have emerged as novel therapeutic agents for PTCLs. In this study, we aimed to explore the immunomodulatory effect of the HDAC inhibitor chidamide on circulating PD-1(+) cells from patients with PTCL, as well as its correlation with treatment response.

**Methods:**

We enrolled newly diagnosed patients with PTCLs treated with a combination of chidamide and chemotherapy. Gene expression profile analysis was performed on peripheral blood PD-1(+) cells, both at baseline and at the end of treatment. A list of differentially expressed genes (DEGs) was identified. Gene Ontology (GO) and Kyoto Encyclopedia of Genes and Genomes (KEGG) pathway analyses were performed to annotate the biological implications of the DEGs. A gene concept network was constructed to identify the key DEGs for further PCR verification.

**Results:**

A total of 302 DEGs were identified in the complete remission (CR) group, including 162 upregulated and 140 downregulated genes. In contrast, only 12 DEGs were identified in the non-CR group. GO analysis revealed that these upregulated DEGs were mainly involved in chemokine activity, cell chemotaxis, and cellular response to interleukin-1 and interferon-γ. Furthermore, KEGG pathway analysis showed that these DEGs were enriched in cytokine-cytokine receptor interaction and chemokine signaling pathways. The innate immune signaling pathways, including the Toll-like and NOD-like receptor signaling pathways, were also influenced. The gene concept network revealed that the key upregulated genes belonged to the C-C chemokine family.

**Conclusion:**

Our results showed that chidamide treatment notably enhanced the expression of genes associated with chemokine activity and chemotaxis function of circulating PD-1(+) cells. By recruiting immune cells and improving the innate immune function of PD-1(+) cells, chidamide may reshape the tumor microenvironment to an anti-tumor phenotype and synergize with checkpoint inhibitors.

## Introduction

Peripheral T-cell lymphomas (PTCLs) are a heterogeneous group of mature T-cell and natural killer (NK) - cell neoplasms, characterized by aggressive clinical behavior and poor prognosis (1, 2). Given the unsatisfactory outcomes of patients with PTCLs upon receiving conventional anthracycline-based chemotherapies, there is a need for novel targeted therapies and immunotherapies. In recent years, histone deacetylase (HDAC) inhibitors have emerged as novel therapeutic agents against PTCLs. The anti-tumor effects of HDAC inhibitors are commonly known as inducing apoptosis and cell cycle arrest (3). Beyond the direct anti-tumor effect, recent advances show that HDAC inhibitors are also involved in the immune-mediated anti-tumor effect (4–6). However, the mechanism is still not clear.

Programmed cell death protein 1 (PD-1), expressed on immune cells, and its ligand PD ligand 1 (PD-L1), expressed on tumor cells, are crucial regulators of the tumor immunosurveillance system (7). The binding of PD-1 to PD-L1 results in suppression of the host immune response and escape of tumor cells from immune surveillance. Previously, we found that the innate immunity of circulating PD-1(+) cells from patients with PTCLs was compromised compared with healthy controls (8). Furthermore, our *in vitro* study revealed that the class I HDAC inhibitor chidamide enhances IFN-γ production and restores the cytotoxic activity of PD-1(+) cells from patients with PTCL, indicating that chidamide may exert its immunomodulatory effect by regulating the function of PD-1(+) cells (8).

Here, we explored the impact of chidamide as an immune regulator on circulating PD-1(+) cells and its correlation with treatment response. Gene expression profile (GEP) analysis was performed on peripheral blood PD-1(+) cells from patients with PTCL, both at baseline and end of first-line treatment. Differentially expressed genes (DEGs) were identified and their biological implications were investigated. The findings described in this study demonstrate a novel mechanism by which HDAC inhibitors regulate PD-1(+) cells and highlight the potential of HDAC inhibitors to synergize with immunotherapies.

## Materials and Methods

### Patients and Treatment

Nine patients who were newly diagnosed with PTCL and treated in Peking Union Medical College Hospital (PUMCH) were enrolled in this study. Histological findings were consistent with PTCLs, according to the World Health Organization (WHO) classification. Histologic subtypes included peripheral T-cell lymphoma, not otherwise specified (PTCL, NOS), anaplastic large cell lymphoma, ALK-negative (ALCL, ALK-), and angioimmunoblastic T-cell lymphoma (AITL).

All patients received a combination of chidamide with cyclophosphamide, epirubicin, vindesine, prednisone, and etoposide (CHOEP) regimen as first-line chemotherapy. The regimen of CHOEP chemotherapy were as follows: cyclophosphamide (750 mg/m^2^ intravenously on day 1), epirubicin (70 mg/m^2^ intravenously on day 1), vindesine (4 mg intravenously on day 1), prednisone (100 mg/d orally on days 1–5), and etoposide (100 mg intravenously on days 1-3). Chidamide 20mg twice weekly was started on day 1 of the first cycle of CHOEP therapy and administered continuously.

Treatment responses were assessed according to the 2014 Lugano classification criteria and classified as complete remission (CR), partial remission (PR), stable disease (SD), and progressive disease (PD) (9). Patients were divided according to the treatment response into CR and non-CR groups (including patients with PR, SD, and PD response). The study was approved by the institutional review board of PUMCH. All participants provided written informed consent.

### Isolation of PD-1(+) Cells

Blood samples were collected both at baseline and at the end of first-line treatment. Flow cytometry analysis was performed to ensure that there were no tumor cells in the peripheral blood. Peripheral blood mononuclear cells (PBMCs) were isolated from blood samples using the Ficoll-Hypaque density centrifugation method. Approximately 10^6^–10^8^ PBMCs were harvested from 25 mL of peripheral blood. Flow cytometry analysis was performed on PBMCs to evaluate the components of PD-1(+) cells. The cells were labelled with anti-PD-1-PE, anti-CD4-APC, anti-CD8-FITC, anti-CD56-PE, and anti-CD14-APC antibodies.

PD-1(+) cells were isolated from PBMCs using the magnetic-activated cell sorting (MACS) method according to the manufacturer’s instructions (Miltenyi Biotech, Germany). Briefly, PBMCs were first incubated with 20 µL anti-CD279-PE antibodies (Miltenyi Biotech, Germany; 130-117-384) at 4°C for 20 min. After washing and centrifugation, the cells were incubated with anti-PE microbeads (Miltenyi Biotech, Germany; 130‐048‐801). The labeled cell suspension was then filled into the reservoir of a large-volume separation column (Miltenyi Biotech, Germany) placed in a magnetic field. After removing the column from the magnetic field, PD-1(+) cells were eluted by flushing the column with PBS buffer. The collected PD-1(+) cell suspension was counted and prepared for mRNA extraction.

### Construction of mRNA Library and Sequencing

Total RNA was extracted from the collected PD-1(+) cells using TRIzol reagent (Invitrogen, Carlsbad, CA, USA, 15596018). A NanoDrop 2000 (Thermo Scientific, USA) was used to verify the purity of the RNA. The quality of RNA was measured using the Qubit RNA Assay Kit in Qubit 2.0 Fluorometer (Life Technologies, USA). The sequencing library of each RNA sample was prepared using an Ion Total RNA-Seq Kit v2 (Life Technologies, USA) according to the manufacturer’s instructions. Emulsion PCR was performed using the cDNA library as template. RNA-sequencing (RNA-Seq) was conducted by NovelBio Bio-Pharm Technology Co. Ltd (Shanghai, China) using an ABI Ion Proton (Life Technologies, USA) instrument.

### Gene Expression Data Analysis

Differentially expressed genes (DEGs), between pre-treatment and post-treatment conditions and between CR and non-CR group at baseline, were filtered using the EBseq algorithm. Genes with an absolute fold change (FC) of > 1.5, or < 0.667, and false discovery rate (FDR) < 0.05, were considered significant DEGs. Average linkage hierarchical clustering was performed, and heatmaps were generated.

### Gene Ontology (GO) and Kyoto Encyclopedia of Genes and Genomes (KEGG) Pathway Analyses

GO analysis was performed to annotate the biological functions of the DEGs, including three GO categories: biological process (BP), cellular component (CC), and molecular function (MF). GO annotations were downloaded from the NCBI (http://www.ncbi.nlm.nih.gov), Gene Ontology (http://www.geneontology.org), and UniProt (http://www.uniprot.org). Pathway annotation of DEGs was performed using the KEGG database. Fisher’s exact test was used to identify the significantly influenced GO categories and KEGG pathways. Statistical significance was set at P < 0.05. The gene concept network was constructed using cluster Profiler for enriched GO biological pathways with statistical significance (P < 0.05) (10).

## Results

### Patient Characteristics

Nine newly diagnosed patients with PTCL, administered chidamide combined with CHOEP regimen as first-line chemotherapy, were included in this study. The CR group included 5 patients and the non-CR group included 4 patients (3 PR and 1 PD). The baseline characteristics of patients in the 2 groups are listed in **Table 1**. The median age at diagnosis was 45 (range, 27–71) years, and the male: female ratio was 4:5. Histologic subtypes included peripheral PTCL, NOS (n = 2), ALK-ALCL (n = 3), and AITL (n = 4). Most patients were in clinical stages III–IV (6/9, 66.7%). The proportion of high-intermediate or high-risk patients, based on the International Prognostic Index (IPI) score, was 2/9 (22.2%).

**Table 1:** Clinical characteristics of the patients in the 2 groups.

Characteristic	CR group (n = 5)	Non-CR group (n = 4)
Age, years		
Median (range)	42 (27-58)	55 (22-71)
Sex, male	2	2
Ann Arbor stage III/IV	3	3
ECOG performance status>1	2	1
IPI score>2	1	1
Histologic subtypes		
PTCL, NOS	1	1
AITL	2	2
ALK- ALCL	2	1

ECOG, Eastern Cooperative Oncology Group; IPI, International Prognostic Index; PTCL, NOS, peripheral T-cell lymphoma; not otherwise specified; AITL, angioimmunoblastic T-cell lymphoma; ALK- ALCL, anaplastic large cell lymphoma, ALK negative.

### Components of PD-1(+) Cells

**Figure 1:**
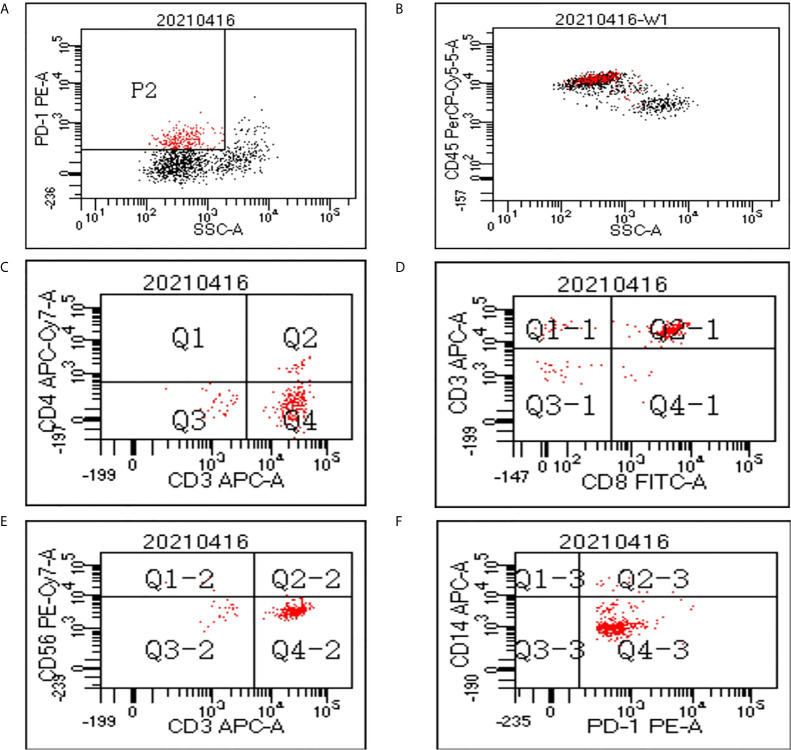
Flow cytometry data of peripheral blood mononuclear cells (PBMCs). **(A)** In the cytogram of side scatter (SSC) versus PD-1, the population P2 refers to the PD-1(+) cells which were colored red. **(B)** The PD-1(+) cells mainly fall in the cluster of lymphocytes in the cytogram of SSC versus CD45. A gate was set to show only the cells in P2 population on the cytogram of CD4 versus CD3 **(C)**, CD8 versus CD3 **(D)**, CD56 versus CD3 **(E)**, and CD14 versus PD-1 **(F)**.

Flow cytometry analysis was performed on PBMCs to evaluate the components of PD-1(+) cells in 3 patients. **Figure 1** shows the flow cytometry result of one patient. The average proportion of PD-1 (+) cells in PBMCs was 18.8%. The average proportion of CD4+, CD8+, CD56+, and CD14+ cells in the PD-1(+) cell subset were 13.3%, 80.9%, 3.2%, and 3.1%, which indicated the proportion of CD4+ T-cells, CD8+ T-cells, NK cells, and monocytes, respectively.

### DEGs Between the Pre-Treatment and Post-Treatment Conditions

**Figure 2:**
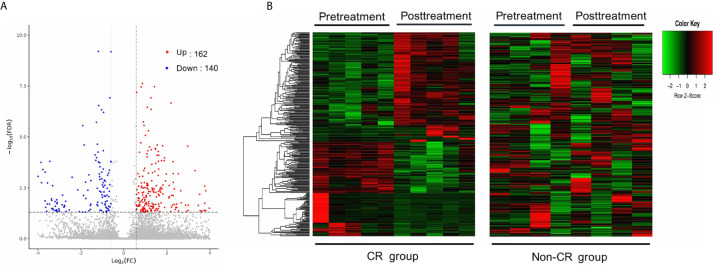
Differentially expressed genes (DEGs) between pre-treatment and post-treatment conditions. **(A)** Volcano plot of DEGs between pre-and post-treatment conditions in the CR group. Significantly up-regulated genes are marked in light red and downregulated ones are marked in blue. **(B)** Hierarchical clustering heatmap. Heatmaps were generated using the expression levels of the 302 DEGs identified in the CR group. The abscissa indicates different samples, and the ordinate indicates different gene probes. These genes were only differentially expressed in the CR group (left), but not in the non-CR group (right).

The gene expression profiles of peripheral blood PD-1(+) cells were compared between the pre-treatment and post-treatment conditions. With a criterion of FDR < 0.05 and |log_2_ FC| ≥ 0.58, a total of 302 DEGs were identified in the CR group, including 162 up-regulated and 140 down-regulated genes (**Figure 2A** **)**. Contrastingly, only 12 DEGs were identified in the non-CR group, including 9 up-regulated and 3 down-regulated genes.

To test whether these DEGs, identified in the CR group, were consistently expressed between the CR and non-CR groups, hierarchical clustering heat maps were generated using the expression levels of the 302 DEGs. These genes were only differentially expressed between pre-treatment and post-treatment conditions in the CR group (**Figure 2B**).

### GO and KEGG Pathway Analyses of DEGs Between the Pre-Treatment and Post-Treatment Conditions

**Figure 3:**
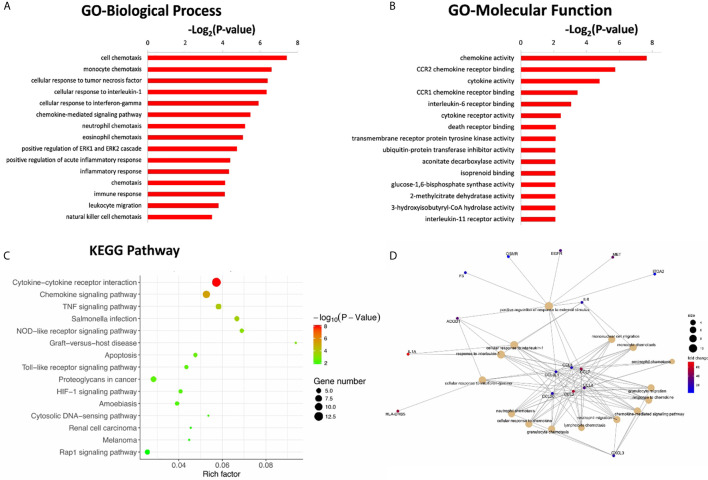
The top 15 significant GO terms for the 162 up-regulated DEGs in the categories of biological process **(A)**, molecular function **(B)**. **(C)** The bubble chart of the top 15 significant KEGG pathways. The number of genes related to each pathway is indicated by different sizes and fold change by different colors. **(D)** Gene concept network of the top 15 GO terms. The number of genes related to each term is indicated by different sizes and fold change by different colors.

To further investigate the potential functional mechanisms, the 302 DEGs identified in the CR group were subjected to GO analysis. The top 15 significant GO terms for the 162 upregulated DEGs in the BP and MF categories are shown in **Figures 3A, B**. GO annotation and significance analysis revealed that these DEGs were mainly involved in chemokine activity, cell chemotaxis (including monocyte, neutrophil, and lymphocyte chemotaxis), cellular response to interleukin-1 (IL-1) and interferon-gamma (IFN-γ).

Pathway annotation of the DEGs was performed using the KEGG database. The top 15 significant KEGG pathways for the 162 upregulated DEGs are shown in **Figure 3C**. Furthermore, pathway analyses also revealed that these DEGs were mainly involved in cytokine-cytokine receptor interactions and chemokine signaling pathways. The innate immune signaling pathways, including Toll-like receptor and NOD-like receptor signaling pathways, were also influenced.

To further explore the interaction of these DEGs and identify key DEGs for further PCR verification, a gene concept network was established based on the top 15 significant GO terms in the BP category. The key up-regulated genes located in the center of the network belonged to the C-C chemokine family, including CCL2, CCL3L1, CCL4, CCL7, CCL8, and CCL20 (**Figure 3D**). The products of these genes recruit monocytes, neutrophils, memory T cells, and dendritic cells (11, 12).

### GO and KEGG Pathway Analyses of DEGs Between CR and Non-CR Group at Baseline

**Figure 4:**
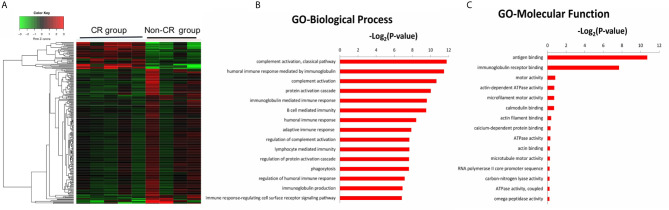
**(A)** Hierarchical clustering heatmap of DEGs between the CR and non-CR group at baseline level. The top 15 significant GO terms for the 470 DEGs in the categories of biological process **(B)**, molecular function **(C)**.

The gene expression profiles of peripheral blood PD-1(+) cells were also compared between the CR and non-CR group at baseline level. A total of 470 DEGs were identified, including 115 up-regulated and 355 down-regulated genes (**Figure 4A** **)**. The 470 DEGs were further subjected to GO analysis. The top 15 significant GO terms in the BP and MF categories are shown in **Figures 4B, C**. GO annotation revealed that these DEGs were mainly involved in humoral immune response, including complement activation and immunoglobulin mediated immune response, which were all down-regulated in the CR group.

## Discussion

PTCLs account for 25–30% of non-Hodgkin’s lymphomas (NHLs) in China, significantly higher than that of western countries (13). Contrasting the progress made in the treatment of B-cell NHLs, the management of patients with PTCL is disappointing, with no major progress made over the past decade. Conventional anthracycline-based regimens remain the frontline regimen for patients newly diagnosed with PTCL. However, except for ALK-positive ALCL, most types of PTCLs exhibit a poor long-term survival of 30–40% (2).

In recent years, new agents, including HDAC and checkpoint inhibitors, have shown some promise for the treatment of PTCLs. At present, the available HDAC inhibitors worldwide include vorinostat, romidepsin, belinostat, and chidamide, an innovative class I HDAC inhibitor, independently developed in China (14). The overall response rates (ORRs) of HDAC inhibitors for patients with relapsed or refractory PTCLs were around 25–39% (15–17). The clinical benefit of PD-1 inhibitors for patients with PTCLs is also being investigated in several clinical trials, with an ORR of approximately 33–40% for patients with relapsed or refractory PTCL (18, 19). A significant limitation of these new agents is the relatively low response rate as monotherapy. Therefore, the combination of such new agents with conventional chemotherapies can potentially be an effective approach, in response to the search for improved treatments for patients with PTCL.

HDAC inhibitors prevent the removal of acetyl groups by HDAC and maintain accessible conformation of chromatin, resulting in increased transcriptional activity of anti-tumor genes (20). In recent studies, HDAC inhibitors have also been shown to have an immune-mediated anti-tumor effect. Both *in vitro* and *in vivo* studies have evaluated the novel role of HDAC inhibitors in manipulating the immune landscape and the potential ability to augment the response to immunotherapy. Guerriero et al. found that the HDAC inhibitor TMP195 promotes macrophage differentiation toward an anti-tumor phenotype in mouse models of breast cancer (4). Zheng et al. found that the HDAC inhibitor romidepsin enhances T cell chemokine expression and promoted T cell infiltration in mouse models of lung cancer, resulting in a strongly augmented response to PD-1 inhibitor (5). Kim et al. found that HDAC inhibitor CG-745 induces prolonged cytotoxic T cell activation and suppresses M2 macrophage polarization, promoting the effect of PD-1 blockade in mouse models of hepatocellular cancer (6).

Despite the relatively widespread use of HDAC inhibitors in PTCLs, their exact role in the immune system of patients with PTCL remains poorly understood. Our results showed that chidamide treatment notably enhanced the expression of genes associated with chemokine activity and chemotaxis function of circulating PD-1(+) cells, which might promote the recruitment of lymphocytes, monocytes, neutrophils, and dendritic cells. These innate immune cells are major components of the tumor microenvironment. In addition, KEGG pathway analysis revealed that the expression of genes involved in the innate immune signaling pathways of PD-1(+) cells was up-regulated after chidamide treatment. By regulating the innate immune cell trafficking and improving the innate immune function of circulating PD-1(+) cells, chidamide may reshape the tumor microenvironment to an anti-tumor phenotype. This may be an attractive approach to anti-tumor immunotherapy.

As suggested by previous studies, insufficient tumor-infiltrating CD8+ T cells and T-cell exhaustion are recognized as major resistance mechanisms to checkpoint inhibitors (21, 22). During the exhausting state of T-cells, cytokine expression is impaired, including interleukin-2, TNF-α, and IFN-γ. Our study found that genes associated with lymphocyte chemotaxis, TNF signaling pathway, and cellular response to IFN-γ were up-regulated after chidamide treatment. By recruiting lymphocytes to the tumor microenvironment and enhancing reinvigoration of exhausted CD8+ T-cells, chidamide treatment may “prime” the host immune response and synergize with checkpoint inhibitors. Pairing chidamide with PD-1 blockade may offer potential benefits in the treatment of patients with PTCLs. Notably, several clinical trials employing this combination approach are now underway. In a multicenter phase Ib/II trial conducted in China, the ORR of chidamide combined with PD-1 inhibitor (sintilimab) for relapsed/refractory extranodal NK/T - cell lymphoma was 58.3%, which was better than either single drug (23).

The up-regulated expression of genes associated with chemotaxis and innate immunity of PD-1(+) cells was only observed in patients in the CR group. Contrastingly, no significant changes were observed in the gene expression of PD-1(+) cells of patients in the non-CR group. This indicated that treatment failure may be partially due to failure of immune reconstitution in patients with PTCLs. When comparing the gene expression of PD-1(+) cells between the CR and non-CR group at baseline level, we found that genes associated with humoral immune response, including complement activation and immunoglobulin mediated immune response, were up-regulated in the non-CR group. The differences of baseline gene expression may be part of the reason for the differences of treatment response. However, based on the current research, it is difficult to explain how these differences of gene expression are related to the efficacy of HDAC inhibitor treatment.

The limitations of this study need to be acknowledged. First, the set of PD-1(+) cells is complex and comprises the following: CD8+ T-cells, CD4+ T-cells, NK cells, and monocytes. Additional studies are required to determine the specific roles of each of these cell types. In future studies, we will perform flow cytometry sorting and single-cell sequencing. Second, our results require verification *via* PCR analysis. In *vitro* cell experiments using transwell assays can also be performed to evaluate the chemotactic function of PD-1(+) cells. Additionally, the synergistic effect of chidamide and PD-1 inhibitors could be further assessed *via* animal experiments. expression pattern may not be related to the type of disease.

In conclusion, we report here for the first time that chidamide treatment enhanced the expression of genes associated with chemokine activity and chemotaxis function of circulating PD-1(+) cells from patients with PTCLs. This novel mechanism of action of chidamide may provide a rationale for therapeutic strategies that combine chidamide with PD-1 inhibitors for patients with PTCLs.

## Data Availability Statement

The data presented in the study are deposited in the GEO repository, the accession number is GSE174537.

## Ethics Statement

The studies involving human participants were reviewed and approved by Peking Union Medical College Hospital review board. The patients/participants provided their written informed consent to participate in this study.

## Author Contributions

CW and SH designed the study. CW, SH, and ML performed the experiment. ML and CC analyzed all the data. WW and WZ helped perform the analysis with constructive discussions. CW wrote the main manuscript. DZ reviewed and revised the manuscript. All authors contributed to the article and approved the submitted version.

## Funding

National Natural Science Foundation of China (NSFC) (No.81970188).

CAMS Innovation Fund for Medical Sciences (CIFMS) (No. 2016-12M-1-001).

Natural Science Foundation of Beijing Municipality (No. 7202154).

## Conflict of Interest

The authors declare that the research was conducted in the absence of any commercial or financial relationships that could be construed as a potential conflict of interest.

## Supplementary Material

Supplementary Table 1 Differentially expressed genes of the CR group.

Supplementary Table 2 Differentially expressed genes of the non-CR group.

Supplementary Data Sheet 1 Differentially expressed genes of one patient who received CHOEP treatment alone.

Supplementary Figure 1 PNG: GO enrichment analysis of DEGs in the CR group.

Supplementary Figure 2 PNG: KEGG enrichment analysis of DEGs in the CR group.
