# Supplementary figures and images for: A Novel Mechanism of Action of Histone Deacetylase Inhibitor Chidamide: Enhancing the Chemotaxis Function of Circulating PD-1(+) Cells From Patients With PTCL
Source: Front Oncol. 2021 Jun 1;11:682436. doi: 10.3389/fonc.2021.682436 (PMC8204089; doi:10.3389/fonc.2021.682436)

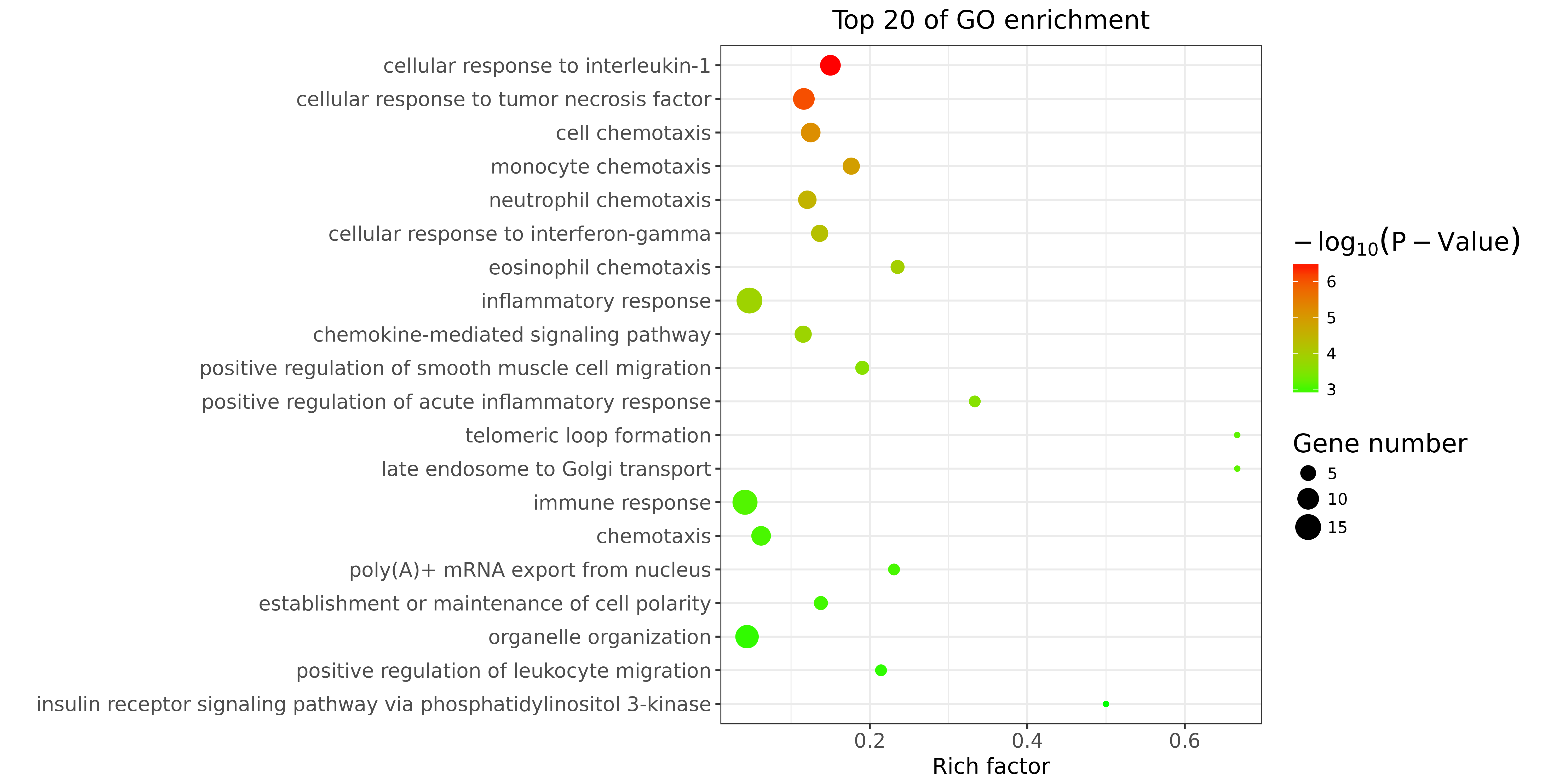

Supplement: Supplementary Figure 1 — PNG: GO enrichment analysis of DEGs in the CR group. [file Image_1.png]

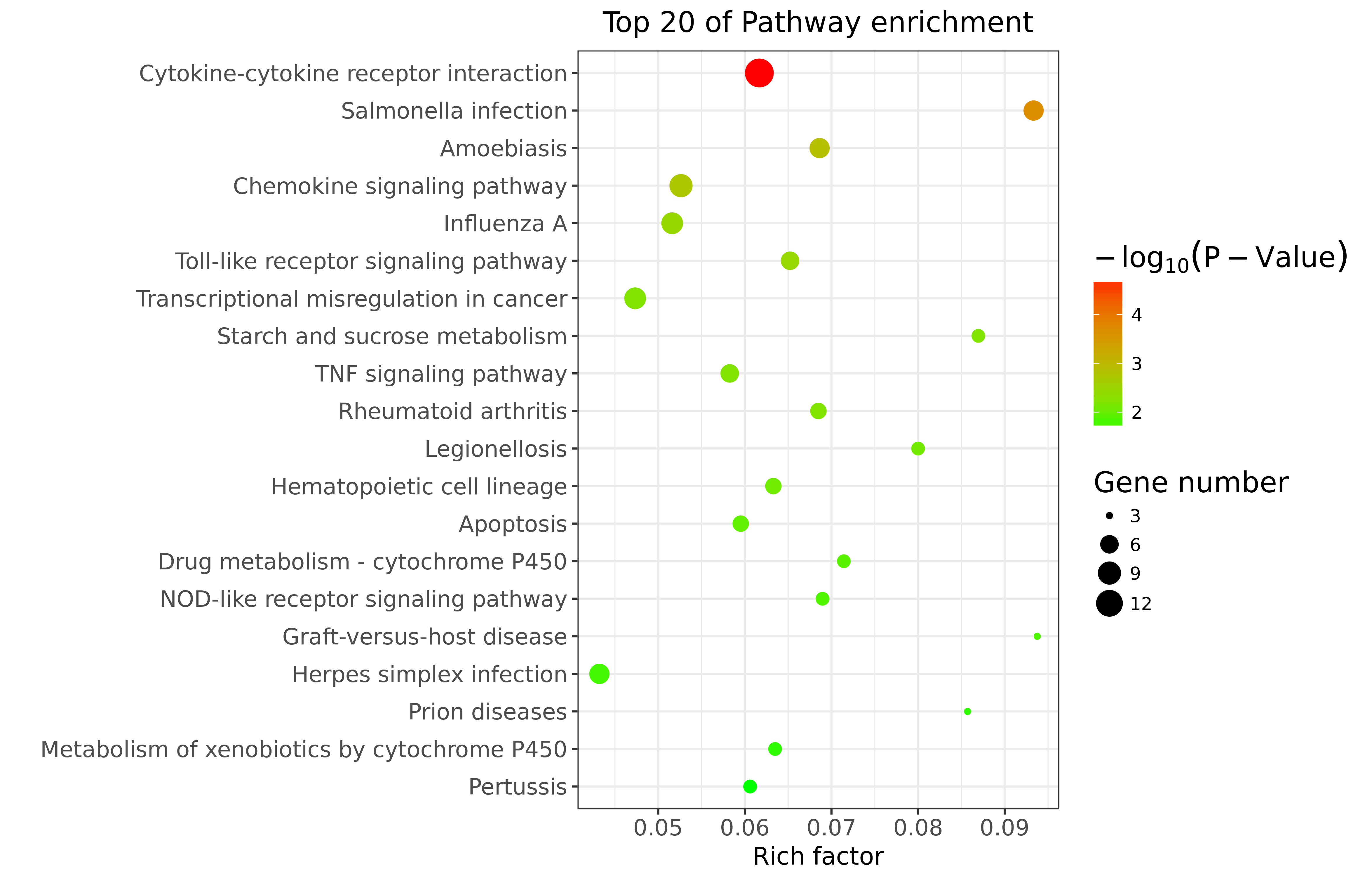

Supplement: Supplementary Figure 2 — PNG: KEGG enrichment analysis of DEGs in the CR group. [file Image_2.png]
